# 5-*O*-Acetyl-d-ribono-1,4-lactone

**DOI:** 10.1107/S1600536811038670

**Published:** 2011-09-30

**Authors:** Adailton J. Bortoluzzi, Damianni Sebrão, Marcus M. Sá, M. G. Nascimento

**Affiliations:** a Depto. de Química - UFSC, 88040-900 - Florianópolis, SC, Brazil

## Abstract

The title compound, C_7_H_10_O_6_, was obtained from a regioselective enzyme-catalysed acyl­ation of d-ribono-1,4-lactone. The five-membered ring of the acyl­ated sugar shows an envelope conformation. In the crystal, the mol­ecules are linked by inter­molecular O—H⋯O hydrogen-bonds, forming a one-dimensional polymeric structure parallel to [010]. In addition, packing analysis shows stacking along the *b* axis.

## Related literature

For general background to carbohydrates, see: Corma *et al.* (2007 ▶); Han *et al.* (1993 ▶); Simone *et al.* (2005 ▶). For biocatalysed acyl­ation reactions, see: Díaz-Rodríguez *et al.* (2005 ▶); Wu *et al.* (2008 ▶). For related structures, see: Shalaby *et al.* (1994 ▶); Bye (1979 ▶); Amador *et al.* (2004 ▶); Sá *et al.* (2008 ▶); Gress & Jeffrey (1976 ▶).
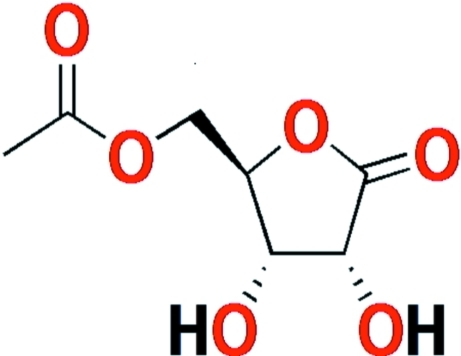


## Experimental

### 

#### Crystal data

C_7_H_10_O_6_
*M* *_r_* = 190.15
Monoclinic, 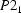

*a* = 6.1409 (4) Å
*b* = 5.1952 (15) Å
*c* = 13.1844 (18) Å
β = 95.118 (12)°
*V* = 418.95 (14) Å^3^
*Z* = 2
Mo *K*α radiation
μ = 0.13 mm^−1^
*T* = 293 K
0.50 × 0.30 × 0.13 mm

#### Data collection

Enraf–Nonius CAD-4 diffractometer
2164 measured reflections
1346 independent reflections
1015 reflections with *I* > 2σ(*I*)
*R*_int_ = 0.046
3 standard reflections every 200 reflections  intensity decay: 1%

#### Refinement

*R*[*F* ^2^ > 2σ(*F* ^2^)] = 0.047
*wR*(*F* ^2^) = 0.135
*S* = 1.07
1346 reflections
127 parameters
1 restraint
H atoms treated by a mixture of independent and constrained refinement
Δρ_max_ = 0.29 e Å^−3^
Δρ_min_ = −0.18 e Å^−3^

### 

Data collection: *CAD-4 Software* (Enraf–Nonius, 1989 ▶); cell refinement: *SET4* in *CAD-4 Software*; data reduction: *HELENA* (Spek, 1996 ▶); program(s) used to solve structure: *SIR97* (Altomare *et al.*, 1999 ▶); program(s) used to refine structure: *SHELXL97* (Sheldrick, 2008 ▶); molecular graphics: *PLATON* (Spek, 2009 ▶); software used to prepare material for publication: *SHELXL97*.

## Supplementary Material

Crystal structure: contains datablock(s) global, I. DOI: 10.1107/S1600536811038670/lr2026sup1.cif

Structure factors: contains datablock(s) I. DOI: 10.1107/S1600536811038670/lr2026Isup2.hkl

Supplementary material file. DOI: 10.1107/S1600536811038670/lr2026Isup3.mol

Additional supplementary materials:  crystallographic information; 3D view; checkCIF report

## Figures and Tables

**Table 1:** Hydrogen-bond geometry (Å, °)

*D*—H⋯*A*	*D*—H	H⋯*A*	*D*⋯*A*	*D*—H⋯*A*
O3—H3⋯O4^i^	0.85 (5)	1.95 (5)	2.781 (3)	164 (3)
O4—H4⋯O2^i^	0.85 (5)	2.15 (5)	2.910 (3)	148 (5)
O4—H4⋯O3^i^	0.85 (5)	2.41 (6)	3.086 (4)	136 (4)

Symmetry code: (i) 
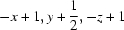
.

**Fig. 1.**
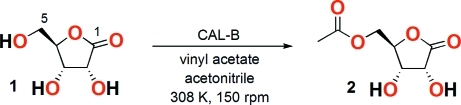
Biocatalyzed acylation reaction.

**Fig. 2.**
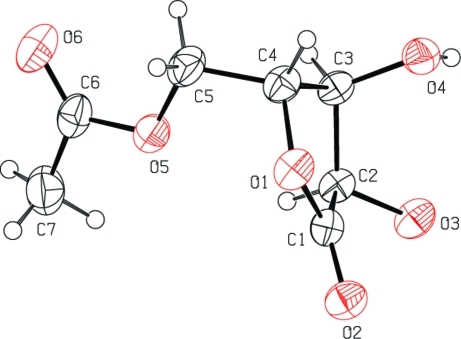
The molecular structure of enantiomeric pair of the title compound showing the atom-labelling scheme. Ellipsoids are drawn at the 40% probability level.

**Fig. 3.**
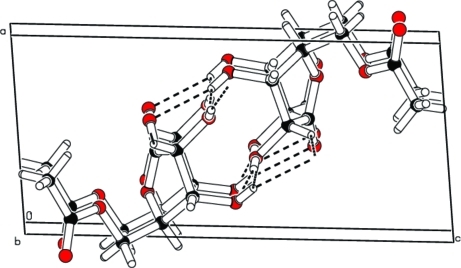
Partial packing of the title compound showing hydrogen bonds.

## supplementary crystallographic information

### Comment

Carbohydrates are valuable sources for the production of synthetic compounds of general relevance (Corma *et al.*, 2007). D-Ribono-1,4-lactone (1) is an inexpensive and abundant sugar derivative that is commercially available from renewable resources (Han *et al.*, 1993, Simone *et al.*, 2005). Many synthetic transformations involving 1 lead to unexpected processes ranging from rearrangements to functional group migrations. In such cases, single-crystal X-ray analysis is the only reliable method for the correct structural and conformational assignments (Sá *et al.*, 2008). Enzyme-catalyzed acylation of sugars is, in general, regioselective. Lipases (EC 3.1.1.3) are the most used biocatalyst for this purpose, especially *Candida antarctica* lipase B - CAL-B (Díaz-Rodríguez *et al.*, 2005; Wu *et al.*, 2008). We describe herein the crystal structure of 5-*O*-acetyl-D-ribono-1,4-lactone (2), synthesized from the regioselective acetylation of 1 using CAL-B (Fig. 1).

The molecular structure of the title compound exhibits its 1,4-lactone ring with envelope conformation, which is enveloped on C3 (Fig. 2). Hydroxyl groups are involved in different types of intermolecular O—H···O hydrogen-bonds (Table 1). Hydroxyl group (O3) is the donor for linear hydrogen-bond (O3—H3···O4), whereas hydroxyl group (O4) is the donor for bifurcated interactions (O4—H4···O2 and O4—H4···O3). These interactions link molecules forming one-dimensional zigzag infinite chain parallel to [010] direction. Also, packing analysis shows stack along the *b* crystallographic axis (Fig. 3).

### Experimental

The reaction was initiated by dissolving D-ribono-1,4-lactone (74.0 mg, 0.5 mmol) and vinyl acetate (0.14 ml, 1.5 mmol) in anhydrous acetonitrile (10.0 ml) followed by the addition of CAL-B (10.0 mg, Novozym 435, 10,000 PLU/g). The mixture was shaken at 308 K and 150 rpm for 24 h. The reaction was stopped by filtering off the lipase. Finally, solvent was evaporated and the product 5-*O*-acetyl-D-ribono-1,4-lactone was obtained as a white solid (94% yield). Careful recrystallization from acetone provided the crystals (413–414 K) suitable for X-ray diffraction analysis.

### Refinement

H atoms attached to carbon atoms were placed at their idealized positions with distances of 0.98, 0.97 and 0.96 Å and *U*_eq_ fixed at 1.2 and 1.5 times *U*_iso_ of the preceding atom for CH, CH_2_ and CH_3_, respectively. H atoms of the hydroxyl groups were found from difference map and treated as free atoms. The final refinement of the structure was done averaging all equivalents.

### Crystal data

**Table:**

C_7_H_10_O_6_	*F*(000) = 200
*M**_r_* = 190.15	*D*_x_ = 1.507 Mg m^−^^3^
Monoclinic, *P*2_1_	Mo *K*α radiation, λ = 0.71073 Å
Hall symbol: P 2yb	Cell parameters from 25 reflections
*a* = 6.1409 (4) Å	θ = 3.5–20.5°
*b* = 5.1952 (15) Å	µ = 0.13 mm^−^^1^
*c* = 13.1844 (18) Å	*T* = 293 K
β = 95.118 (12)°	Prismatic, colorless
*V* = 418.95 (14) Å^3^	0.50 × 0.30 × 0.13 mm
*Z* = 2	

### Data collection

**Table:**

Enraf–Nonius CAD-4 diffractometer	*R*_int_ = 0.046
Radiation source: fine-focus sealed tube	θ_max_ = 30.0°, θ_min_ = 1.6°
graphite	*h* = −8→8
ω–2θ scans	*k* = −7→2
2164 measured reflections	*l* = −18→2
1346 independent reflections	3 standard reflections every 200 reflections
1015 reflections with *I* > 2σ(*I*)	intensity decay: 1%

### Refinement

**Table:**

Refinement on *F*^2^	Primary atom site location: structure-invariant direct methods
Least-squares matrix: full	Secondary atom site location: difference Fourier map
*R*[*F*^2^ > 2σ(*F*^2^)] = 0.047	Hydrogen site location: inferred from neighbouring sites
*wR*(*F*^2^) = 0.135	H atoms treated by a mixture of independent and constrained refinement
*S* = 1.07	*w* = 1/[σ^2^(*F*_o_^2^) + (0.0807*P*)^2^ + 0.0065*P*] where *P* = (*F*_o_^2^ + 2*F*_c_^2^)/3
1346 reflections	(Δ/σ)_max_ < 0.001
127 parameters	Δρ_max_ = 0.29 e Å^−^^3^
1 restraint	Δρ_min_ = −0.18 e Å^−^^3^

### Fractional atomic coordinates and isotropic or equivalent isotropic displacement parameters (Å^2^)

**Table:**

	*x*	*y*	*z*	*U*_iso_*/*U*_eq_	
C1	0.4264 (4)	−0.0865 (5)	0.32056 (19)	0.0355 (5)	
C2	0.4464 (4)	0.1808 (5)	0.3674 (2)	0.0344 (5)	
H2	0.4839	0.3062	0.3162	0.041*	
C3	0.2154 (4)	0.2326 (5)	0.3976 (2)	0.0364 (6)	
H3A	0.1774	0.4155	0.3913	0.044*	
C4	0.0742 (4)	0.0687 (6)	0.3213 (2)	0.0405 (6)	
H4A	−0.0460	−0.0050	0.3559	0.049*	
C5	−0.0206 (5)	0.2048 (8)	0.2270 (2)	0.0507 (8)	
H5A	−0.1029	0.0850	0.1820	0.061*	
H5B	−0.1181	0.3413	0.2448	0.061*	
C6	0.1100 (5)	0.5012 (7)	0.1095 (2)	0.0482 (7)	
C7	0.3079 (6)	0.5894 (10)	0.0629 (3)	0.0660 (11)	
H7A	0.3294	0.4848	0.0046	0.099*	
H7B	0.4329	0.5748	0.1118	0.099*	
H7C	0.2895	0.7658	0.0422	0.099*	
O1	0.2157 (3)	−0.1406 (4)	0.29314 (16)	0.0433 (5)	
O2	0.5706 (3)	−0.2355 (4)	0.30942 (17)	0.0480 (5)	
O3	0.6139 (3)	0.1709 (5)	0.44808 (17)	0.0449 (5)	
O4	0.1877 (3)	0.1377 (5)	0.49711 (16)	0.0445 (5)	
O5	0.1590 (3)	0.3103 (5)	0.17745 (16)	0.0482 (6)	
O6	−0.0705 (4)	0.5837 (6)	0.0916 (2)	0.0666 (8)	
H3	0.661 (6)	0.325 (9)	0.455 (3)	0.047 (10)*	
H4	0.265 (7)	0.229 (12)	0.540 (4)	0.070 (14)*	

### Atomic displacement parameters (Å^2^)

**Table:**

	*U*^11^	*U*^22^	*U*^33^	*U*^12^	*U*^13^	*U*^23^
C1	0.0392 (12)	0.0292 (12)	0.0375 (12)	−0.0038 (11)	0.0002 (10)	0.0020 (11)
C2	0.0301 (10)	0.0282 (12)	0.0445 (13)	−0.0040 (10)	0.0004 (9)	0.0007 (11)
C3	0.0332 (11)	0.0304 (14)	0.0452 (13)	0.0003 (10)	0.0004 (9)	−0.0002 (11)
C4	0.0320 (11)	0.0398 (16)	0.0489 (14)	−0.0059 (12)	−0.0006 (10)	0.0034 (13)
C5	0.0368 (12)	0.060 (2)	0.0535 (16)	−0.0001 (15)	−0.0068 (11)	0.0076 (17)
C6	0.0556 (16)	0.0446 (16)	0.0417 (14)	0.0035 (16)	−0.0107 (12)	−0.0012 (14)
C7	0.068 (2)	0.078 (3)	0.0513 (18)	−0.002 (2)	0.0015 (16)	0.015 (2)
O1	0.0416 (9)	0.0331 (10)	0.0535 (11)	−0.0079 (9)	−0.0054 (8)	−0.0032 (9)
O2	0.0480 (11)	0.0389 (12)	0.0566 (12)	0.0027 (10)	0.0018 (9)	−0.0052 (10)
O3	0.0371 (9)	0.0401 (13)	0.0555 (12)	−0.0052 (10)	−0.0078 (8)	−0.0051 (10)
O4	0.0411 (9)	0.0487 (13)	0.0438 (10)	0.0013 (10)	0.0033 (8)	−0.0021 (10)
O5	0.0429 (10)	0.0534 (14)	0.0478 (11)	0.0049 (10)	0.0014 (8)	0.0081 (11)
O6	0.0569 (13)	0.0666 (18)	0.0728 (15)	0.0090 (13)	−0.0140 (11)	0.0155 (15)

### Geometric parameters (Å, °)

**Table:**

C1—O2	1.195 (3)	C5—O5	1.439 (4)
C1—O1	1.342 (3)	C5—H5A	0.9700
C1—C2	1.521 (4)	C5—H5B	0.9700
C2—O3	1.413 (3)	C6—O6	1.192 (4)
C2—C3	1.531 (4)	C6—O5	1.352 (4)
C2—H2	0.9800	C6—C7	1.482 (5)
C3—O4	1.425 (4)	C7—H7A	0.9600
C3—C4	1.528 (4)	C7—H7B	0.9600
C3—H3A	0.9800	C7—H7C	0.9600
C4—O1	1.460 (4)	O3—H3	0.85 (5)
C4—C5	1.502 (4)	O4—H4	0.85 (5)
C4—H4A	0.9800		
			
O2—C1—O1	122.6 (3)	C3—C4—H4A	108.5
O2—C1—C2	127.4 (2)	O5—C5—C4	107.4 (2)
O1—C1—C2	110.0 (2)	O5—C5—H5A	110.2
O3—C2—C1	107.4 (2)	C4—C5—H5A	110.2
O3—C2—C3	116.1 (2)	O5—C5—H5B	110.2
C1—C2—C3	102.9 (2)	C4—C5—H5B	110.2
O3—C2—H2	110.0	H5A—C5—H5B	108.5
C1—C2—H2	110.0	O6—C6—O5	122.9 (3)
C3—C2—H2	110.0	O6—C6—C7	126.1 (3)
O4—C3—C4	107.8 (2)	O5—C6—C7	111.0 (3)
O4—C3—C2	111.6 (2)	C6—C7—H7A	109.5
C4—C3—C2	102.4 (2)	C6—C7—H7B	109.5
O4—C3—H3A	111.5	H7A—C7—H7B	109.5
C4—C3—H3A	111.5	C6—C7—H7C	109.5
C2—C3—H3A	111.5	H7A—C7—H7C	109.5
O1—C4—C5	109.6 (3)	H7B—C7—H7C	109.5
O1—C4—C3	105.5 (2)	C1—O1—C4	110.9 (2)
C5—C4—C3	116.0 (3)	C2—O3—H3	105 (2)
O1—C4—H4A	108.5	C3—O4—H4	108 (3)
C5—C4—H4A	108.5	C6—O5—C5	116.4 (2)

### Hydrogen-bond geometry (Å, °)

**Table:**

*D*—H···*A*	*D*—H	H···*A*	*D*···*A*	*D*—H···*A*
O3—H3···O4^i^	0.85 (5)	1.95 (5)	2.781 (3)	164 (3)
O4—H4···O2^i^	0.85 (5)	2.15 (5)	2.910 (3)	148 (5)
O4—H4···O3^i^	0.85 (5)	2.41 (6)	3.086 (4)	136 (4)

Symmetry codes: (i) −*x*+1, *y*+1/2, −*z*+1.
